# *Vibrio cholerae* accessory colonisation factor AcfC: a chemotactic protein with a role in hyperinfectivity

**DOI:** 10.1038/s41598-018-26570-7

**Published:** 2018-05-30

**Authors:** Esmeralda Valiente, Cadi Davies, Dominic C. Mills, Maria Getino, Jennifer M. Ritchie, Brendan W. Wren

**Affiliations:** 1 Department of Pathogen Molecular Biology, London School of Hygiene and Tropical Medicine, Keppel Street, WC1E 7HT London, UK; 2 Robert Frederick Smith School of Chemical and Biomolecular Engineering, Cornell University, Olin Hall, Ithaca, NY USA; 3 Department of Microbial Sciences, Faculty of Health and Medical Sciences, University of Surrey, Guildford, GU2 7XH UK

## Abstract

*Vibrio cholerae* O1 El Tor is an aquatic Gram-negative bacterium responsible for the current seventh pandemic of the diarrheal disease, cholera. A previous whole-genome analysis on *V. cholerae* O1 El Tor strains from the 2010 epidemic in Pakistan showed that all strains contained the *V. cholerae* pathogenicity island-1 and the accessory colonisation gene *acfC* (VC_0841). Here we show that *acfC* possess an open reading frame of 770 bp encoding a protein with a predicted size of 28 kDa, which shares high amino acid similarity with two adhesion proteins found in other enteropathogens, including Paa in serotype O45 porcine enteropathogenic *Escherichia coli* and PEB3 in *Campylobacter jejuni*. Using a defined *acfC* deletion mutant, we studied the specific role of AcfC in *V. cholerae* O1 El Tor environmental survival, colonisation and virulence in two infection model systems (*Galleria mellonella* and infant rabbits). Our results indicate that AcfC might be a periplasmic sulfate-binding protein that affects chemotaxis towards mucin and bacterial infectivity in the infant rabbit model of cholera. Overall, our findings suggest that AcfC contributes to the chemotactic response of WT *V. cholerae* and plays an important role in defining the overall distribution of the organism within the intestine.

## Introduction

*Vibrio cholerae* O1 El Tor is an aquatic, Gram-negative, single flagellated bacterium responsible for the current seventh cholera pandemic^1^. After entering the human body by ingestion of contaminated food or water, bacteria migrate towards the surface of the small intestine. Colonisation of the intestine leads to the release of cholera toxin (CT) and the development of profuse, watery diarrhoea that is characteristic of the disease. Cholera is treatable with oral rehydration therapy, but if left untreated, can quickly lead to severe dehydration, toxic shock, and death in less than 24 hours post infection^2^. Cholera remains an important global health problem, mainly affecting areas with poor water sanitation. Each year cholera affects an estimated 3 million people, resulting in over 100,000 deaths, particularly young children^3^, causing considerable adverse economic consequences to countries in Asia, Africa, Oceania and South America^4^.

CT and the toxin co-regulated pilus (TCP) are well established as the main virulence factors of *V. cholerae*^5–7^. CT is responsible for the profuse watery diarrhoea that characterises the disease^6^ whereas TCP, a type IV pilus, is essential for *V. cholerae* colonisation of the small intestine, as demonstrated in both human volunteers^8^ and animal models^9^. A myriad of other factors has also been associated with *V. cholerae* pathogenicity^10^. Of these, motility and chemotaxis have been the subject of considerable study given their proposed role in controlling the intestinal localisation of the organism. Historically Freter and colleagues proposed that chemotactic-based motility was responsible for the preferential localisation of *V. cholerae* to the crypts of the distal small intestine^10,11^. However, recent studies^12,13^ suggest that the distribution of *Vibrio cholerae* in the intestine is as a result of a complex interplay between motility, chemotaxis and host mucin. Chemotaxis is controlled by numerous inner membrane-localised methyl-accepting chemotaxis proteins (MCPs) and soluble chemotaxis proteins^11,12^. However, at the molecular level, the chemotaxis network is incompletely understood. Chemotaxis also has a role in the aquatic environment, where it allows bacteria to find suitable environmental and host surfaces, made, for example, of chitin. Chitin - a polymer of N-acetylglucosamine, the second most abundant polymer in nature - is present in the exoskeleton of crustaceans and serves as both a carbon source and as a chemoattractant of marine *Vibrio* species^14,15^.

An important genomic island involved in cholera disease is the *V. cholerae* pathogenicity island 1 (VPI-1), which contains the TCP gene cluster. VPI-1 also possesses a cluster of four accessory colonisation factor (*acf*) genes, designated as *acfA, acfB, acfC* and *acfD*^16^. Both TCP and *acf* genes are regulated by ToxR, the cholera toxin transcriptional activator^17^. The *acf* genes in *V. cholerae* O395 (O1 Classical biotype) appear to be required for efficient intestinal mice colonisation and biogenesis of the toxin-associated pilus of *V. cholerae*. In addition, AcfB was shown to be an inner membrane protein that affects motility in *V. cholerae* O395^17^. However, in the case of *V. cholerae* O1 El Tor, *acf* genes do not seem to have a role in virulence.

In August 2010, Pakistan experienced the worst flooding in its recorded history, affecting more than 20 million people^18^, followed by a cholera epidemic^19^. A genetic analysis of *V. cholerae* O1 El Tor strains from the floods identified two distinct groups, isolated from different geographic regions: Pakistan subclades 1 (PSC-1) (isolates from the coastal city of Karachi) and 2 (PSC-2) (in countrywide areas). Amongst other differences, genome analyses showed that PSC-2 strains had a frame-shift mutation in *acfC* introducing several stop codons, whereas *acfC* encoded a full open reading frame in PSC-1 strains^19^. PSC-1 strains appear to be replacing PSC-2 strains in Pakistan (Nick Thomson, Wellcome Trust Sanger Institute, Cambridge, personal communication). In addition, comprehensive data base searching revealed that AcfC-like proteins are only found in two other bacterial species, which are both enteropathogens (*Campylobacter jejuni* and *Escherichia coli)*, suggesting a new family of proteins that maybe important for survival in the mammalian gut. We therefore hypothesized that the presence of a functional *acfC* in *V. cholerae* may confer an advantage to strains in PSC-1 to survive in the environment and/or to colonise the human small intestine.

In this work, we investigated the biological role of AcfC in *V. cholerae* O1 El Tor and found that it might be a periplasmic sulfate-binding protein, which affects chemotaxis towards mucin and has a direct impact on bacterial hyperinfectivity in the infant rabbit model of disease.

## Results

### Sequence analysis of AcfC suggests a periplasmic localisation and reveals high identity to PEB3 and Paa proteins

**Figure 1:**
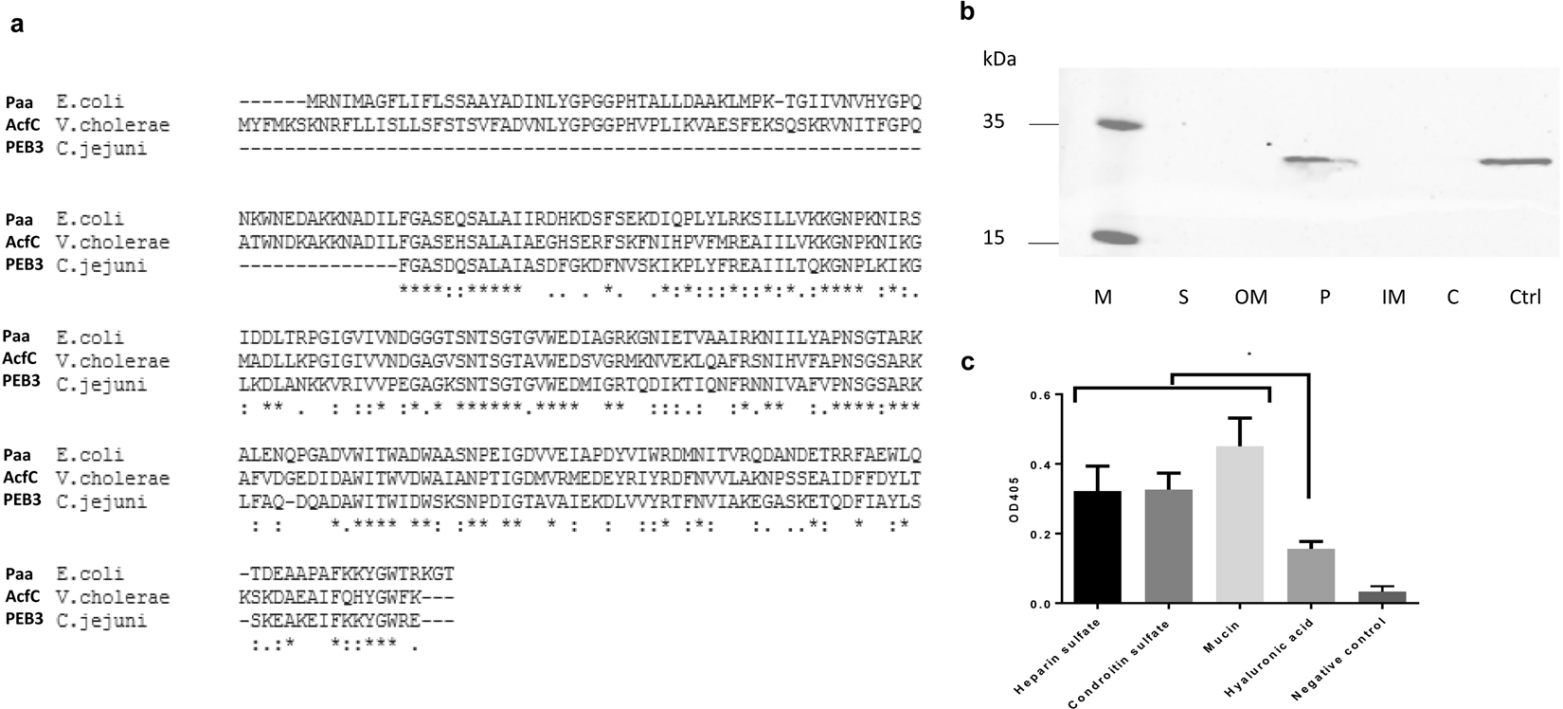
(**a**) Blast alignments of *V. cholerae* AcfC with similar proteins from enteropathogenic bacteria (Paa from *E. coli* and PEB3 from *C. jejuni*) using Clustal W software; (**b**) Detection of *V. cholerae* AcfC expression in the different bacterial cell compartments using anti-AcfC antibodies. M: Page ruler plus pre-stained protein ladder (Thermo Fisher Scientific); S: Supernatant fraction; OM: Outer membrane fraction; P: Periplasmic fraction; IM: Inner membrane fraction; C: Cytoplasmic fraction; Ctrl: Ni-NTA purified His_6_-tagged AcfC, used as a positive control; (**c**) ELISA analysis of binding AcfC to different sulfated glycosaminoglycans (heparin sulfate and chondroitin sulfate), non-sufated glycosaminoglycan (hyaluronic acid) and type II porcine mucin. The values are the means ± standard deviation (n = 3). *p < 0.05, as determined by Tukey’s test.

The *V. cholerae acfC* is an open reading frame of 770 bp encoding a polypeptide of 256 amino acids that is predicted to be localised to the periplasm, according to the Protein Subcellular Localization Prediction for Gram-negative Bacteria (http://www.csbio.sjtu.edu.cn/bioinf/Cell-PLoc-2/). A protein blast search (https://blast.ncbi.nlm.nih.gov) indicated that AcfC protein has high similarity to *E. coli* Paa and *C. jejuni* PEB3. The amino acid sequences of AcfC, PEB3 and Paa were aligned by ClustalW (Fig. 1a). AcfC has 48,4% identity to PEB3 and 47% identity to Paa. After cleavage of the first 24 amino acids, which likely encodes a signal sequence, the mature AcfC protein is predicted to have a size of 28 kDa (http://www.uniprot.org/uniprot). We confirmed the periplasmic localisation of AcfC by cellular fractionation and western blot with anti-AcfC antibodies (Fig. 1b).

### *V. cholerae* AcfC is a sulfate-binding protein

The presence of lysine- and arginine-rich regions in the amino acid sequence suggests AcfC may have sulfate-binding properties. We thus tested the ability of His_6_-tagged AcfC to bind to plastic surfaces coated with sulfated proteins - such as heparin sulfate, chondroitin sulfate and porcine mucin type II or the non-sulfated protein hyaluronic acid as a negative control. Using an ELISA assay, we found that His_6_-tagged AcfC bound to both heparin, chondroitin sulfate and mucin, but significantly less to hyaluronic acid (Fig. 1c). These results suggest that that AcfC might prefer binding to sulfate-containing proteins (Fig. 1c).

### AcfC is not involved in Caco-2 or HT-29MTX-E12 cell adhesion

**Figure 2:**
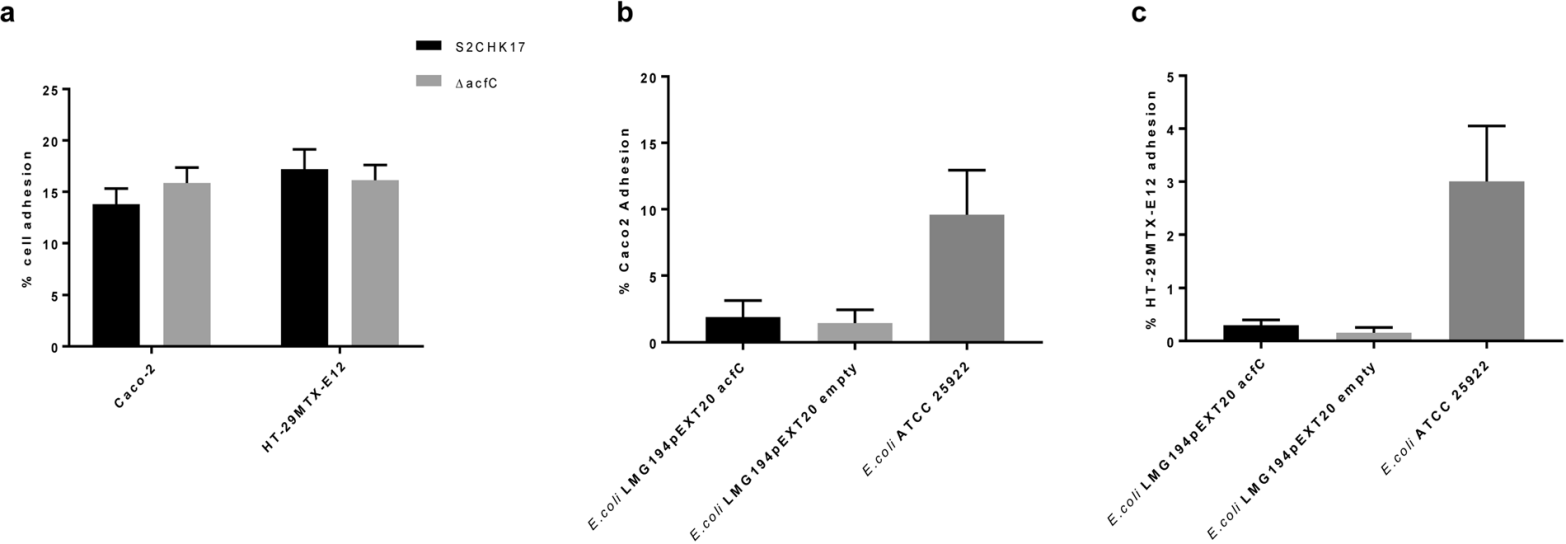
(**a**) Adhesion of *V. cholerae* S2CHK17 and *ΔacfC* to Caco-2 and HT-29MTX-E12 cell lines. Adhesion of LMG194 and LMG194 with pEXT20-AcfC construct to Caco-2 (**b**) and HT-29MTX-E12 (**c**) cell lines. *E. coli* ATCC25922 was used as an adhesion positive control. Each value is the average of three replicates. Error bars represent the standard deviation (n = 3). Statistical analyses using the Tukey’s multiple comparisons test showed no significant differences in adhesion to cell lines.

Given that orthologous AcfC proteins are involved in epithelial cell adhesion, we tested if that was also the case for AcfC in *V. cholerae*. The role of AcfC in adhesion of *V. cholerae* was assayed using Caco-2 cells, a cell line well known to produce comparatively low amounts of mucin. The adherence of wild type (S2CHK17 strain) and *V. cholerae* Δ*acfC* to Caco-2 cells was similar (Fig. 2a). We next repeated the experiment with a previously described mucin hyper-producer cell line, HT-29MTX-E12 cells. However, we found no differences in adherence between *V. cholerae* wild type (S2CHK17 strain) and *V. cholerae* Δ*acfC* (Fig. 2a). These findings indicate that AcfC, unlike its protein homologues (PEB3 or/and Paa)^20,21^, is not involved in cell adhesion in the conditions of our experiment.

To confirm this result, we investigated whether recombinant expression of AcfC in a non-adherent *E. coli* strain (LMG194) would be able to alter its’ adhesion properties. In line with our results obtained with *V. cholerae*, expression of His_6_-tagged AcfC in *E. coli* LMG194 did not alter adhesion to Caco-2 or HT-29MTX-E12 cells compared to the empty vector control (Fig. 2b and c). The positive control strain, *E. coli* ATCC25922, however adhered effectively to Caco-2 cells (10% ± SD) and HT-29MTX-E12 cells (3%±) indicating the validity of the assay. Based on these results, we concluded that AcfC is not an adhesion to intestinal cell lines.

### Biofilm formation

**Figure 3:**
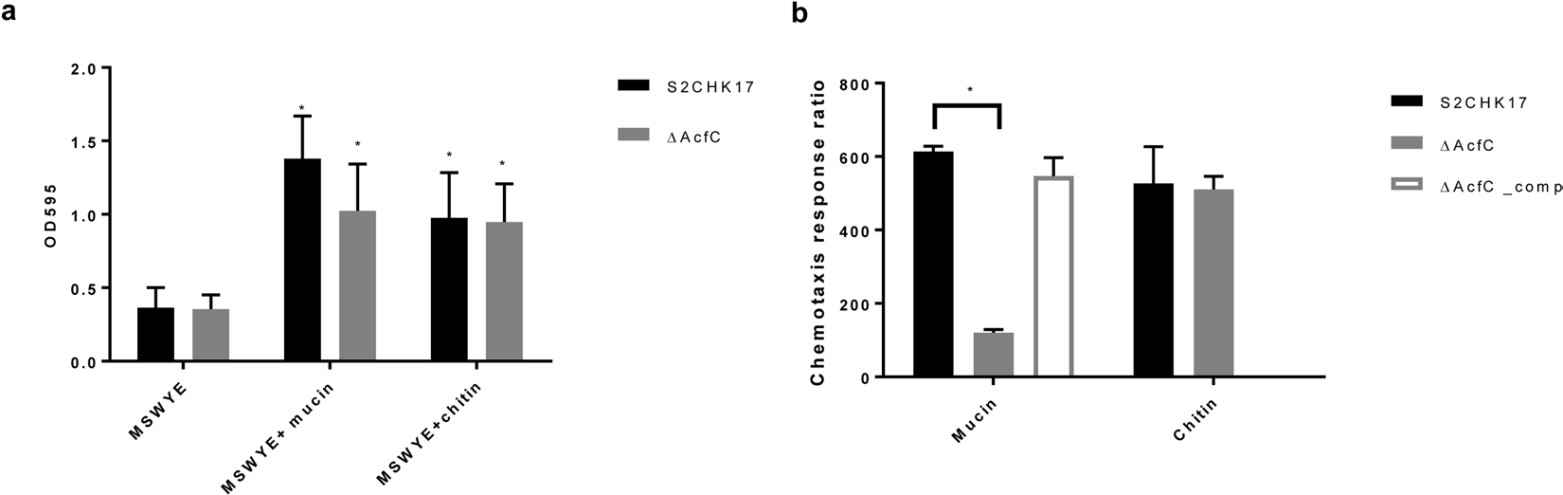
*V. cholerae* S2CHK17 and *ΔacfC* biofilm formation (**a**), chemotaxis towards mucin including AcfC complement strain and chemotaxis towards chitin (**b**). The values are the means ± standard deviation (n = 3). *p < 0.05, as determined by Tukey’s test.

To survive and colonise both the aquatic environment and the human intestine, *V. cholerae* forms biofilms on different abiotic and biotic surfaces. We thus studied the ability of *V. cholerae* S2CHK17 and Δ*acfC* to form biofilms on 24-well plates with or without mucin and/or chitin. Our results demonstrate that the presence of mucin or chitin enhances biofilm formation in both *V. cholerae* S2CHK17 and *ΔacfC*. However, the absence of AcfC does not affect biofilm formation neither on mucin nor on a chitin surface (Fig. 3a).

### AcfC mediates chemotaxis to mucin but not chitin

The initial stages of colonisation of the human intestinal epithelium often involve the efficient movement and taxis towards mucin covering epithelial cells. We studied bacterial chemotaxis by filling a syringe with the chemoattractant, chitin or mucin, and inserting it into a pipette tip containing a bacterial suspension. We found that *V. cholerae ΔacfC* is significantly less chemotactic towards mucin compared to the wild type strain; complementation of the *acfC* gene restored normal chemotaxis (Fig. 3b). However, we found no significant differences in chemotaxis towards chitin between *V. cholerae* wild type and Δ*acfC* mutant (Fig. 3b). Our results suggest that AcfC is required for chemotaxis towards mucin, but does not affect chemotaxis towards chitin.

### AcfC deletion does not affect flagellar assembly or motility

**Figure 4:**
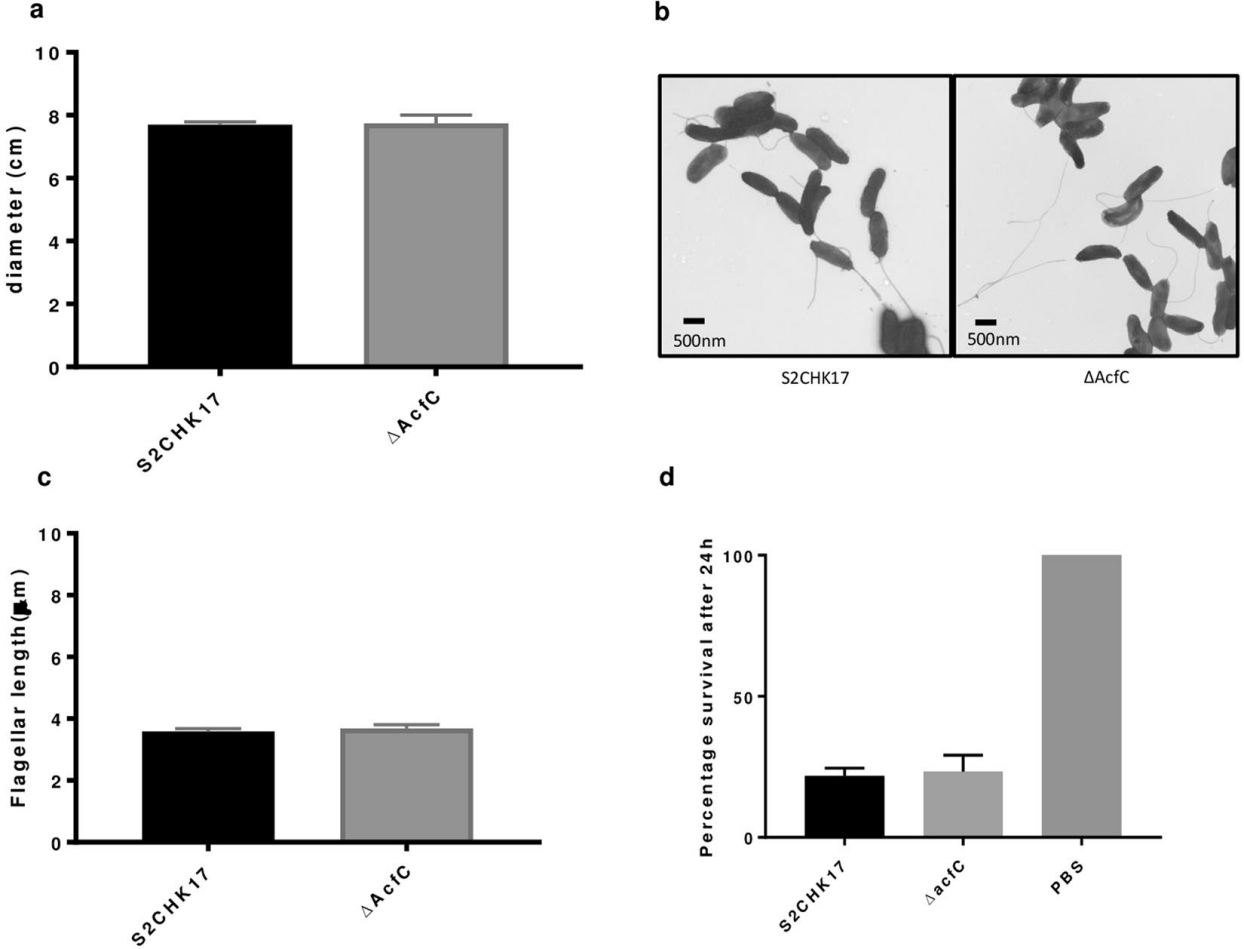
(**a**) *V. cholerae* S2CHK17 and *ΔacfC* motility; (**b**) TEM pictures; (**c**) Flagella measurements: The flagella length of one hundred bacteria were measured in each strain and TEM images were analysed using the line measuring tool of Image J 1.50i software (National Institutes of Health, USA); (**d**) *G. mellonella* infection results Representative data of survival rate of 3 biological replicates of 10 individual *G. mellonella* injected with 2.6 ± 1.0 × 10^5^ CFU of each strain in 10 µl of sterilised PBS and incubated at 37 °C. Survival was assayed by response to touch or discoloration. Killing by WT and *ΔacfC* was observed after 24 h. No killing was observed in the PBS injection control for the length of the experiment. Error bars represent the standard deviation (n = 3). Statistical analyses using the Tukey’s multiple comparisons test showed no significant differences in *V. cholerae* survival.

To rule out that the attenuated chemotactic phenotype in the *ΔacfC* mutant was not due to a defect on motility or flagella assembly, we measured *V. cholerae* motility and flagellar length. Both wild type *V. cholerae* and the *ΔacfC* mutant were similarly motile (Fig. 4a) and bacteria of both strains possessed a single polar flagellum (Fig. 4b) of similar length (Fig. 4c), suggesting that AcfC has no direct role in motility or flagellar assembly.

### AcfC does not contribute to virulence in *Galleria mellonella*

*G. mellonella* larvae have been used previously as a simple, invertebrate model to study the virulence of different bacterial pathogens, including *V. cholerae*^22^. Pathogenic potential, as reflected by larvae mortality, has been found to correlate with virulence established using mammalian models for several pathogens^23,24^. Injection of *G. mellonella* with 2.6 ± 1.0 × 10^5^ CFU of WT or *Δ*AcfC lead to similar mortality of the wax moth larvae after 24 hours infection (22% versus 24% survival in WT and *Δ*AcfC, respectively; Fig. 4d). Consistent with this finding, the corresponding LD_50_ of the WT and the *ΔacfC* mutant was 1.1 ± 0.3 × 10^5^ CFU per larvae, respectively. As expected, control larvae injected with PBS maintained 100% survival throughout the length of the virulence assay. These results demonstrate that AcfC deletion in *V. cholerae* El Tor has no effect on pathogenesis in the *G. mellonella* model.

### AcfC mutant exhibits increased infectivity and colonisation of the proximal small intestine of infant rabbits

To further explore the virulence of the Δ*acfC* mutant and correlate the response seen in *G. mellonella* larvae to that of a mammalian host, we used the infant rabbit model of cholera^25^. Oral infection of rabbits with ~1 × 10^9^ CFU of WT bacteria caused diarrhoea or intestinal fluid accumulation in ~67% of animals (Table 1), similar to that found previously for other *V. cholerae* O1 El Tor strains^25^, although this was evident by 12 h rather than 18 h post infection. By contrast, the Δ*acfC* mutant caused watery diarrhoea in all (100%) infected rabbits. Furthermore, the rabbits appeared sicker with a trend towards a more rapid and severe infection compared to those given WT organisms. While fluid accumulation was evident in the mid to distal regions of the small intestine of WT-infected animals, this extended proximally into the upper third of the small intestine of animals infected with Δ*acfC* mutant. Moreover, the cecal fluid accumulation ratio (FAR), a surrogate measure for diarrhoea, tended to be greater in rabbits infected with Δ*acfC* mutant compared to WT (Table 1). Thus, overall the Δ*acfC* mutant appeared to exhibit increased (or hyper) infectivity compared to the WT strain.

**Table 1:** Diarrhoeal status of rabbits infected with wild type *V. cholerae* or the Δ*acfC* mutant.

Bacterial strain	WT	Δ*acfC*
Diarrhoea (%)	67	100
**Diarrhoea score** ^**a**^		
Severe	10	19
Mild	2	0
None	6	0
Total no. of animals	18	19
P value^b^	0.008	
FAR^c^	1.51 ± 0.68	2.09 ± 0.96
P value^d^	0.09	

^a^Number of rabbits with diarrhoea as described in the text.

^b^Fisher’s exact test was used to compare the number of WT-infected rabbits with disease versus the ΔacfC mutant.

^c^Fluid accumulation ratio (FAR) is calculated from the weight of the cecal fluid to the tissue for each animal.

^d^Students 2-sided T test.

**Figure 5:**
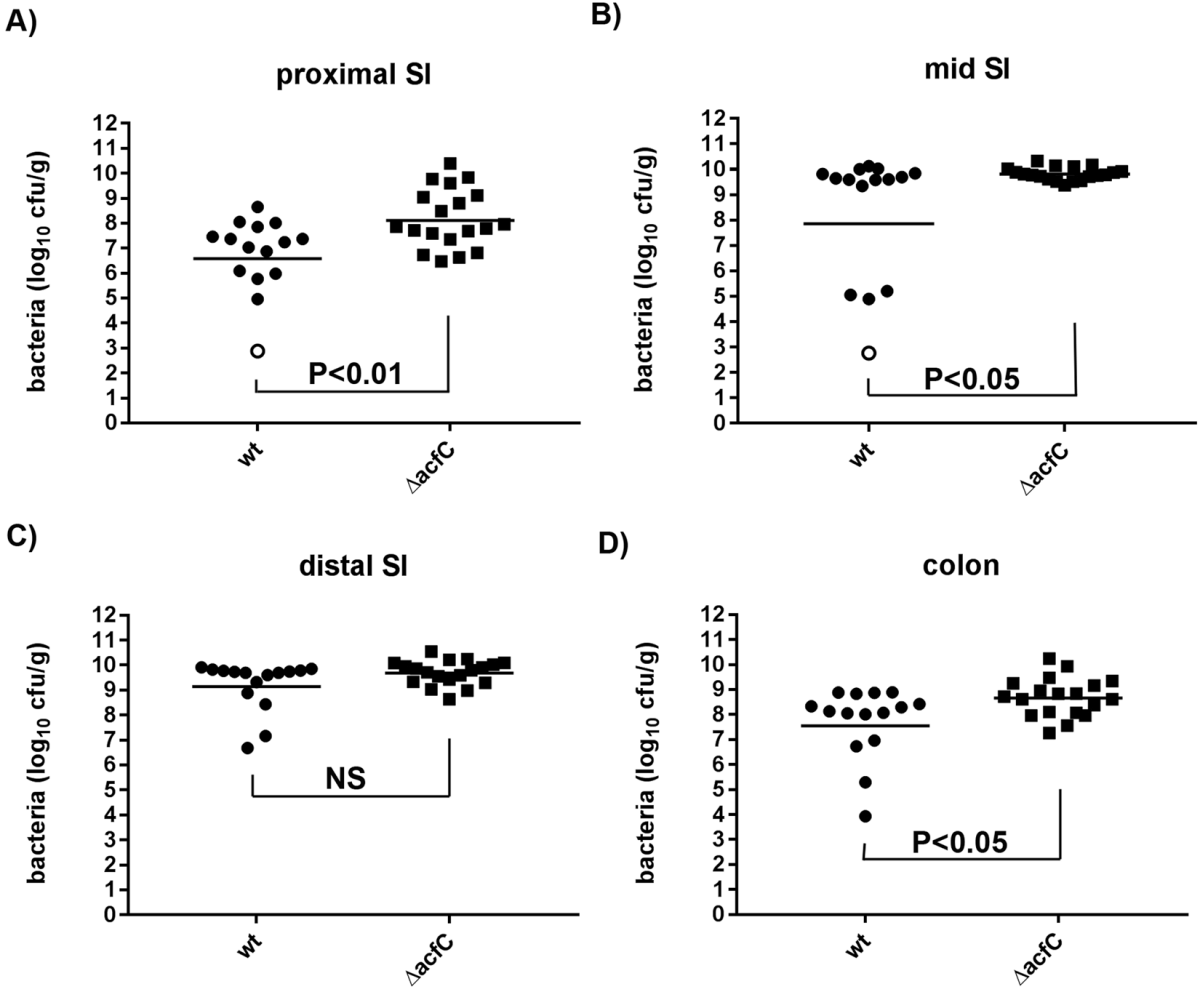
Recovery of WT *V. cholerae* or the Δ*acfC* mutant from different regions of the rabbit intestine at 12 hours post infection. Numbers of CFU recovered from tissue homogenates of different regions of the small intestine (SI) or colon. Open symbols represent samples where the number of recovered bacteria is below the detection limit (value has been set at detection limit). Bars represent the geometric mean and each data point represents an individual animal (WT – 15 rabbits; *acfC* mutant – 19 rabbits; 3 independent litters used for each strain). Data were log transformed and compared using an unpaired 2-tailed Students T test.

Hyperinfectivity of *V. cholerae* has previously been associated with non-chemotactic smooth swimming strains that are better able to colonise the entire length of the small intestine^2,11^. In order to investigate whether enhanced colonisation occurred in rabbits infected with the ΔAcfC mutant, we determined the number of WT and Δ*acfC* mutant bacteria present in tissue homogenates taken from the proximal, mid and distal regions of the small intestine, and from the mid colon of infected rabbits. Higher numbers of the Δ*acfC* mutant were recovered in each of the intestinal sections taken from these single strain infected animals, reaching statistically significant differences in the proximal (P < 0.01) and mid (P < 0.05) small intestine, and the colon (P < 0.05) (Fig. 5). In the tissue samples, ~1 log more bacteria were recovered in *acfC*-infected rabbits than in those given WT *V. cholerae*. Furthermore, significantly higher numbers of the mutant were recovered in the cecal fluid (WT – 3.6 × 10^7^ CFU/mL versus Δ*acfC* mutant 3.6 × 10^8^ CFU/mL; P < 0.05), consistent with the higher burden of *V. cholerae* present in tissue homogenates.

## Discussion

In this study, we provide a detailed characterisation of the *V. cholerae* O1 El Tor accessory colonisation factor, AcfC, which might be a sulfate-binding protein that enhances chemotaxis towards intestinal mucin and contributes to promoting the ‘correct’ localisation of WT *V. cholerae* in the small intestine. As mucus is rich in sulfated molecules^26^ we hypothesize that AcfC might act as a ‘sulfate sensor’ of *V. cholerae* O1 El Tor that senses intestinal mucus and facilitates penetration of the mucus layer by directing chemotaxis towards sulfated molecules at the intestinal surface. The *V. cholerae acfC* gene is part of a polycistronic operon downstream of *acfB* in Vibrio pathogenicity island I. Moreover, *acfB* gene is under the control of ToxR-ToxT regulatory cascade and is structurally and functionally related to methyl-accepting chemotaxis enteric protein^27,28^. It has been suggested that *V.cholerae* AcfB and AcfC interaction activates a chemotaxis signal transduction cascade mediated by Che proteins^29^. It has also been reported that AcfB is another accessory colonisation factor, which affects chemotaxis but does not affect *V. cholerae* virulence in mice. This may be due to overlapping or redundant chemotaxis roles with other proteins, such as toxin co-regulated pilus (TcpI)^28^. Two orthologues of AcfC, PEB3 from *C. jejuni* and Paa from porcine-pathogenic *E.coli* are considered to be adhesins involved in attachment to human epithelial cells^26,30^. However, our work suggests that the primary role of AcfC in *V. cholerae* is that of a chemotaxis-related protein, rather than being directly involved in adhesion to cell lines. AcfC has been also hypothesized to be a secreted protein in *V. cholerae* O395 (O1 El Classical biotype)^30^. By contrast, we confirmed the periplasmic localisation of AcfC in *V. cholerae* O1 El Tor using cellular fractionation and immunoblotting. In *C. jejuni*, PEB3 is also known to be a periplasmic glycoprotein^30^.

AcfC mediates chemotaxis towards mucin but does not have a role in mucin or chitin induced biofilm formation. However, other proteins in *V. cholerae* with a role on chitin induced biofilm formation such as the toxin co-regulated pilus, which mediates bacterial interactions during biofilm formation on chitinaceous surfaces have been described^31^.

Motility is necessary for chemotaxis in *V. cholerae*, but mutants in motility and chemotaxis behave differently in *vivo*^12^. *V. cholerae* chemotaxic mutants exhibited enhanced colonisation in infant mice particularly in the proximal intestine^13^. Our AcfC mutant has an altered chemotaxis phenotype and increased infectivity with enhanced fluid accumulation in AcfC-infected rabbits comparing to wild-type infected animals. All these data are in accord with other non-chemotactic “smooth swimming” mutants which have also increased infectivity^11,12^. In contrast, Kamp and colleagues reported that *acf* genes in *V. cholerae* O1 El Tor are not important in pathogenesis, dissemination and transmission^32^. However, in the study of Kamp *et al*., they sampled the distal small intestine of infant rabbits and did not investigate the other parts of the small intestine, which might explain their contrasting results.

We studied the role of AcfC in virulence by using two *in vivo* model systems. In our *G. mellonella* infection model, AcfC mutant has no effect on pathogenesis. This insect model may only be suitable for crude virulence screening of strains. However, for colonisation studies, an animal model with a more developed intestine would be more appropriate, such as the case of the infant rabbit model.

Taken together, this is the first study showing the accessory colonisation factor AcfC of *V. cholerae* O1 El Tor might act as a periplasmic sulfate-binding protein, affecting bacterial chemotaxis towards mucin. Furthermore, our results show that AcfC has a significant role in governing *V. cholerae* distribution in the small intestine of infant rabbits. It is intriguing to speculate why PSC-2 strains, which lack a functional *acfC* gene, are less prominent in the Pakistan cholera outbreak than PSC-1 strains, given that the former may exhibit increased infectivity. Perhaps despite their increased infectivity, other genetic or phenotypic differences between the lineages aid survival outside of the host, an important aspect of the *V. cholerae* lifecycle.

## Methods

### Bacterial strains and growth conditions

Strains, plasmids and oligonucleotides used in this study are listed in Table 2. *Vibrio cholera*e S2CHK17 and *acfC* mutant were routinely grown on Luria agar (LA), broth (LB) or thiosulfate citrate bile salts sucrose agar (TCBS) at 37 °C. All cultures were grown from glycerol stocks in aerobiosis. *Escherichia coli* DH5α were used for cloning experiments. *E. coli* MFD λ-pir was used as a conjugation donor*. E. coli* LMG194 was used as host strain for protein expression. Where appropriate media was supplemented with ampicillin (100 μg/ml) or chloramphenicol (30 μg/ml).

**Table 2:** Strains, plasmids and oligonucleotides used in this study.

Strain, plasmid and oligonucleotides	Relevant characteristic (s) or sequence (5′ to 3′)	Source or reference
**Strains**		
*Escherichia coli*		
DH5α	F– Φ80*lac*ZΔM15 Δ(*lac*ZYA-*arg*F) U169 *rec*A1 *end*A1 *hsd*R17 (rK–, mK+) *pho*A *sup*E44 λ– *thi*-1 *gyr*A96 *rel*A1	Invitrogen
LMG194	F^−^ Δ*lacX74 galE thi rpsL* Δ*phoA* (*Pvu*II) Δ*ara714 leu*::Tn*10*	Invitrogen
MFD λ-pir	MG1655 RP4-2-Tc::[ΔMu1::aac(3)IV-ΔaphA-Δnic35-ΔMu2::zeo] ΔdapA::(erm-pir) ΔrecA	^43^
*Vibrio cholerae*		
S2CHK17	*V.cholerae* O1 El Tor, isolated from Pakistan 2010	^19^
AcfC mutant	S2CHK17 ΔAcfC, in frame deletion mutant	This study
Plasmids		
pEXT20	Expression vector, Amp^r^, IPTG-inducible	^38^
pDS132	Suicide plasmid, Cn^r^	^35^
pEXT20-AcfC	pEXT20 with optimized RBS and AcfC open reading frame (xxxbp)	This study
pDS132-acfC	pDS132 with SOE PCR fragment (xxxbp)	This study
Oligonucleotides		
VC0841A-SacI	AAGAGCTCTAATGCTGAAGCCATTGCATC	This study
VC0841B	TGTACTCTCCCTAAAATCACTTAAATGATAAACTTACTGATTAAATCATC	This study
VC0841C	GTGATTTTAGGGAGAGTACATGTACTC	This study
VC0841D-SacI	TTGAGCTCATCTGCTTAACTTTGGTAACTGG	This study
Internal primer F	TACAAATTGTTGATATCACAAATCAAGTAG	This study
Internal primer R	CAAAATAAAATAGTAATGCAAAGTGATAAAG	This study
VC0841RBS_EcoRIF	AAGAATTCAGGAGGTAAAACATATGTATTTCATGAAAAGTAAGAATCG	This study
VC0841RBS_XbaIR	TTTCTAGATCAATGATGATGATGATGATGCTTAAACCAGCCATAGTGCT	This study

### *Vibrio cholerae* cell fractionation and AcfC antibody production

*V. cholerae* cells were fractionated following the protocol as described^33^. Each protein fraction was run on a Nu-Page 4–12% Bis-Tris SDS-PAGE gel and analysed by immunoblotting using anti-AcfC antibodies. Experiments were performed in three separate biological replicates and measured as two technical replicates each. AcfC rabbit polyclonal antiserum was obtained from CovalAB UK (Cambridge, UK) against the following peptides: CAESFEKSQSKRVNIT and CFDYLTKSKDAEAIFQHY.

### Expression of C-terminally His_6_-tagged AcfC in *Escherichia coli* and *Vibrio cholerae*

The *acfC* ORF with an optimized ribosomal binding site (RBS) and a C-terminally His_6_-tag were amplified using primer VC0841RBS_EcoRIF and VC0841RBS_XbaI (Table 2) and Phusion polymerase (NEB). The amplicon was cloned into pEXT20 using EcoRI and XbaI cloning sites producing pEXT20-acfC. This construct was transformed into the expression strain *E. coli* LMG194 (Invitrogen) and *V. cholerae* S2CHK17. *E. coli* or *V. cholerae* cells were grown until the OD_600_ was 0.5–0.7. Protein expression was induced by adding isopropyl β-D-1-thiogalactopyranoside (IPTG) at 1 mM for 3 h and 24 h in *E. coli* and *V. cholerae*, respectively. Cultures were centrifuged and pellets were stored at −20 °C. Cells were subsequently thawed on ice, lysed and recombinant protein was purified with Ni-NTA agarose (QIAGEN). Protein samples were run on a Nu-Page 4–12% Bis-Tris SDS-PAGE gel and analysed by immunoblotting using anti-His_6_ antibodies (Abcam, UK).

### Western blot

Proteins were transferred to a nitrocellulose membrane using iBLOT system (Life Technologies). A rabbit anti-Histag (ab9108, Abcam,UK) or anti-acfC antibody were used as primary antibodies and a fluorescently labelled goat anti-rabbit IR Dye 680RD secondary antibody (Li-cor) was used as secondary antibody. The membrane was visualized using an Odyssey fluorescent imager (LI-COR). The un-cropped image of Fig. 1b is in Supplementary Figure 1.

### Sulfate binding assay

Flat-bottomed 96-well plates were coated with 200 µl of 100 µgml^−1^ glycosaminoglycans (chondroitin sulfate and heparin sulfate), type II porcine mucin (Sigma) and non-glycoaminoglycan (hyaluronic acid), at 4 °C for 18 h. The wells were washed with PBS and blocked with 0.5% bovine serum albumin (BSA) for 1 h at 37 °C. The wells were washed and then incubated with 0–200 pmol purified AcfC (in PBS) for 1 h at room temperature and then incubated with 200 µl of rabbit anti-AcfC antibodies (1:1000) in 0.1% BSA in PBS for 1 h at RT. After several washes, bound AcfC was then incubated with alkaline phosphatase conjugated goat anti-rabbit antibody (Sigma, UK) in 0.1% BSA in PBS. Reactions were developed with 50 mM p-nitrophenylphosphate (Sigma, UK). The reactions were stopped by adding 3 M NaOH to each well and absorbance at 405 nm was measured. Coating efficiency of the glycoaminoglycans and non-glycoaminoglycans and porcine mucin used in this study, was analyzed by measuring the protein concentration in each well after PBS washes using a Bradford assay. Wells without coating were used as negative controls. Experiments were performed in three separate biological replicates, measured as three technical replicates each.

### Allelic exchange mutagenesis and complementation

In-frame deletion mutants were constructed using the splicing by overlap extension (SOE) PCR and allelic exchange^34^. We used the *V. cholerae* S2CHK17 genome sequence as a template. Primers are listed in Table 2. The SOE PCR fragment was cloned into the suicide vector pDS132^35^ and electroporated into the *E. coli* MFD λ-pir strain (a diaminopemilic acid (DAP) auxotroph) and then conjugated into *V. cholerae* λ-pir via cross streaking on LB plates containing 0.3 mM diaminopimelic acid (DAP). Growth from these plates was then transferred to LB plates containing 30 μg/ml chloramphenicol to select for *V. cholerae* with the suicide vector only. Double-crossover deletion mutants were then screened by PCR using flanking primers (Table 2) and confirmed by colony PCR and sequencing. The complemented strain was made by electroporation (1.8 kV) of the pEXT20-acfC construct to *acfC* mutant strain. The complemented strain was grown in LB with the corresponding antibiotic and 1 mM IPTG induction for 24 h.

### Cell adhesion to Caco-2 and HT-29MTX-E12 mucus secreting cell lines

The low mucin producer, human Caco-2 colon adenocarcinoma cell line^36^ was obtained from the National Type Culture Collection. The human HT-29MTX-E12 mucus secreting cell line^37^ was obtained from Sigma, UK. The cells were maintained in Gibco RPMI 1640 (Life technologies, USA) or Dulbecco’s modified Eagle’s high glucose medium (Sigma-Aldrich, UK) respectively, both supplemented with 10% (v/v) fetal calf serum, 1% (v/v) non-essential amino acids and 1% (v/v) penicillin-streptomycin (Sigma-Aldrich) in a 5% CO_2_ humidified atmosphere at 37 °C. *Escherichia coli* LMG194 and *V. cholerae* were grown in LA from a glycerol stock for 20 h at 37 °C and then LB was inoculated with a single colony and grown 20 h at 37 °C. A secondary culture was inoculated, and protein expression was induced by adding IPTG 1 mM for 24 h at 37 °C.

For adhesion assay, medium was replaced with 1 ml of 1 × 10^8^ CFU/ml of *E. coli* LMG194 or *V. cholerae* suspended in the previously described antibiotic free tissue culture media (human Caco-2 colon adenocarcinoma and HT-29MTX-E12 mucus secreting cell line with Gibco RPMI 1640 (Life technologies, USA) or Dulbecco’s modified Eagle’s high glucose medium (Sigma-Aldrich, UK) respectively, both with 1% (v/v) fetal calf serum and 1% (v/v) non-essential amino acids). The adhesion assay was performed for 2 h. In parallel, we grew *E.coli* and *V. cholerae* in the antibiotic free tissue culture media we used for the adhesion assay (described above) and checked AcfC expression after 2 h and we were able to detect AcfC expression in *V. cholerae* wild type and *E.coli* with pEXT20-AcfC construct. This could be due to the pTac promoter on the medium copy pEXT20 vector which does lead to leakiness^38^. However, we did not detect AcfC expression in the *acfC* mutant or pEXT20 empty vector.

For adhesion assays, the medium was replaced with antibiotic free medium supplemented with 1% (v/v) fetal calf serum and 1% (v/v) non-essential amino acids 24 hours prior to experiments. Both cell lines were seeded at 2.5 × 10^5^ cells/ml into 24-well plates (Corning Glass Works, Netherlands) using 1 ml of cell suspension per well.

Adhesion assays were performed on Caco2 or HT-29MTX-E12 monolayers that had reached 80–90% confluence in 24-well plates. Infections were carried out for 2 h. For infections, medium was replaced with 1 ml of 1 × 10^8^ CFU/ml of *E. coli* LMG194 or *V. cholerae* suspended in the previously described antibiotic free tissue culture media. After co-incubation, the monolayers were washed 3 times with 1 ml of PBS 1X and incubated with 200 µl of 0.25% trypsin EDTA to break up the monolayer which was subsequently diluted and plated onto LB agar with or without corresponding antibiotic to calculate CFU/ml. *E. coli* ATCC25922 was used as adhesion positive control. Experiments were performed in three separate biological replicates, measured as three technical replicates each.

### Motility and electron microscopy (EM)

Bacteria were grown on LA plates for 24 h, at 37 °C. One colony was inoculated in LB with 0.3% agar (BD Bacto-agar) and incubated at 37 °C for 18 h. Photographs were captured after the incubation time with a Canon 600D SLR. The diameter of the halo was also determined. Experiments were performed in three separate biological replicates, measured as three technical replicates each.

For *V. cholerae* visualization by EM, strains were grown in LB tubes at 37 °C, overnight under aerobic conditions. Each strain was grown in a total volume of 1 ml and was incubated for 18 h. 500 µl of 2.5% paraformaldehide/2.5% glutaraldehyde/0.1 M Na cacodylate pH 7.4 was added to 500 µl of the bacterial culture. Five ml were placed onto a platform coated 300 mesh copper grid for 1 min. The sample was then stained with 10 µl of 0.3% phosphotungstic acid (PTA) pH 7 for 1 min. The PTA was drained and the grid was air-dried before examining on the Jeol 1200EX Transmission Electron Microscope. Digital images were recorded using a side-mounted AMT 2K CCD Digital camera supplied by Deben UK Ltd, IP30 9QS.

### Chemotaxis towards mucin and chitin

Chitin (Sigma, UK) and Type II mucin from porcine stomach (Sigma, UK) was prepared freshly for each assay by suspending at 2% (wt/vol) in chemotaxis buffer (sterile PBS 1X with 0.01 mM filter sterilised EDTA). *V. cholerae* cells were grown to an OD_600_ of between 0.4 and 0.6 in LB broth. Chemotaxis was measured following the protocol as described^39^. Results were compared to control needles containing only chemotaxis buffer to determine the chemotaxis response. The chemotaxis response was calculated as the ratio of test CFU/ml to control CFU/ml. Comparisons were made between strains using at least three biological assays containing two technical replicates each.

### Biofilm formation

*V. cholerae* strains were grown in Marine Sea Water Yeast Extract (MSWYE) agar and broth^40^ overnight at 37 °C. Two ml of culture was inoculated into low evaporation 24-well plates at 1:200 dilution. The cultures were grown statically for one day after which the supernatant was carefully removed and the wells washed with 1 ml of PBS 1X. Each well was incubated with 1% crystal violet (wt/v) at room temperature for 20 min. The crystal violet was dissolved with methanol for 15 min at room temperature. The OD_595_ was measured in a Spectrophotometer (Biotek, UK). Experiments were performed in three separate biological replicates, measured as three technical replicates each.

### Galleria mellonella virulence assay

This assay was performed as previously described^41,42^ using a micro-injection technique whereby 10 µl *V. cholerae* was injected into the haemocoel via the right foreleg, using a Hamilton syringe. *G. mellonella* larvae were bred in sterile conditions at 37 °C. After injection of bacteria, caterpillars were incubated at 37 °C and survival and macroscopic appearance were recorded at 24 h post-infection. Caterpillars were considered dead when they were nonresponsive to touch. Three biological replicates of ten individual *G. mellonella* were performed.

### Infant rabbit infections

All experimental protocols were approved by the local Animal Welfare and Ethical Review Body, the Home Office and carried out in accordance with the UK Animals (Scientific Procedures) Act 1986.

Adult New Zealand White females were obtained from a commercial breeder (Harlan Laboratories, UK) and time-mated when required to produce litters of infant rabbits. Individual litters, typically containing 6–8 pups, were housed as a group in a nest box with the lactating doe for the duration of the experiments. Experiments were performed generally as described previously^25^. Briefly, two- to three day old infant rabbits were treated with ranitidine hydrochloride (GlaxoSmithKline) by intraperitoneal injection (2 mg/kg bodyweight) 2 hours prior to infection. Bacteria were given orally by gavage using a Size 5 French catheter (Arrow International). Infant rabbits were infected with ~1 × 10^9^ CFU WT *V. cholerae* or the Δ*acfC* mutant. The inoculum was prepared from stationary phase (18 h) cultures of the bacteria grown in LB broth supplemented with 100 µg/mL streptomycin at 37 °C with shaking (250 rpm). To prepare the inoculum, bacterial cultures were centrifuged (5 min, 8 rpm) to pellet the cells and the supernatant was removed. Cell pellets were resuspended in sodium bicarbonate solution (2.5 g in 100 mL; pH 9) adjusted to yield a final cell density of ~2 × 10^9^ CFU/mL. Diarrhoea was scored using the following scale: none (rabbits were dry with no signs of faecal contamination or wetness on their ventral surfaces; upon dissection, the colon contained digesta that appeared normal (dark green, hard and formed)); mild (soft yellow stools and/or limited areas of wetness were evident on the rabbits’ fur; upon dissection, digesta was missing from the colon or appeared yellow, soft and unformed), and severe (rabbits exhibited extensive areas of wetness on their tails’ and ventral surfaces; upon dissection no digesta was found in the colon and the cecum and small intestine contained large quantities of clear fluid). Infant rabbits were anesthetized with inhalational isoflurane prior to euthanasia by cervical dislocation.

At euthanasia, intestinal samples and cecal fluid were collected as described previously^25^. The number of *V. cholerae* CFU in tissue samples was determined after homogenisation in PBS by plating on LB supplemented with 100 µg/mL streptomycin. The detection limit of the assays was ~100 CFU/g. Rabbits, which contained no WT *V. cholerae* colonies in any of the intestinal samples were considered ‘uncolonised’ and excluded from subsequent analysis; the reasons why we fail to recover any colonies in a small number (<1 in 10) of rabbits orally-infected with wild type bacteria are unknown. However, rabbits, in which viable *V. cholerae* were detected in at least one intestinal sample, were included in order to capture the natural variation of colonisation and disease that occurs in this host. In these animals, samples that yielded no bacteria at the lowest dilution tested were included in the calculation of the mean values presented in Fig. 5 by using the lower limit of detection as a value.

### Statistical analysis

Data were expressed as mean ± standard deviation. The statistical test used was Tukey’s multiple comparisons test using Prism software 9 version 4.0 (GraphPad Software, Inc, San Diego, CA). p > 0.05 was considered statistically significant. The diarrhoeal state of the rabbits were compared using Fisher’s exact test. Cecal fluid accumulation ratios (FARs) and colonisation data (after log transformation) were statistically analysed using unpaired 2-sided Student T test (GraphPad Prism, San Diego, CA).

### Data availability statement

The datasets generated during and/or analysed during the current study are available from the corresponding author on reasonable request.

## Electronic supplementary material

Supplementary information
