# Supplementary material for: Vibrio cholerae accessory colonisation factor AcfC: a chemotactic protein with a role in hyperinfectivity
Source: Sci Rep. 2018 May 30;8:8390. doi: 10.1038/s41598-018-26570-7 (PMC5976639; doi:10.1038/s41598-018-26570-7)
Supplement: Supplementary file 1 — Supplementary information [file 41598_2018_26570_MOESM1_ESM.pdf]

Supplementary information*: Vibrio cholerae* accessory colonisation factor AcfC: a chemotactic protein with a role in hyperinfectivity

Esmeralda Valiente[1], Cadi Davies[1], Dominic C. Mills[2], Maria Getino[3], Jennifer M. Ritchie[3] and Brendan W. Wren[1*]

[1] Department of Pathogen Molecular Biology. London School of Hygiene and Tropical Medicine. Keppel Street, WC1E 7HT, London, UK.

[2] Robert Frederick Smith School of Chemical and Biomolecular Engineering, Cornell University, Olin Hall, Ithaca, NY, USA

[3] Department of Microbial Sciences, Faculty of Health and Medical Sciences, University of Surrey, Guildford GU2 7XH, UK

*Correspondence address: email: Brendan.wren@lshtm.ac.uk

Department of Pathogen Molecular Biology, LSHTM, Keppel Street, London, WC1E 7HT, UK

Phone: +44 (0) 2079272288; Fax: + 44 (0) 207 6374314

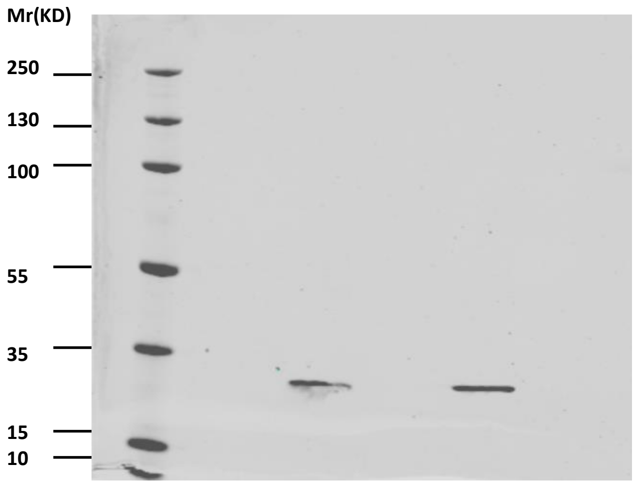

Supplementary figure 1. Un-cropped blot Figure 1b
